# Supplementary figures and images for: Genome-wide identification and characterization of heat shock protein family 70 provides insight into its divergent functions on immune response and development of Paralichthys olivaceus
Source: PeerJ. 2019 Nov 11;7:e7781. doi: 10.7717/peerj.7781 (PMC6855204; doi:10.7717/peerj.7781)

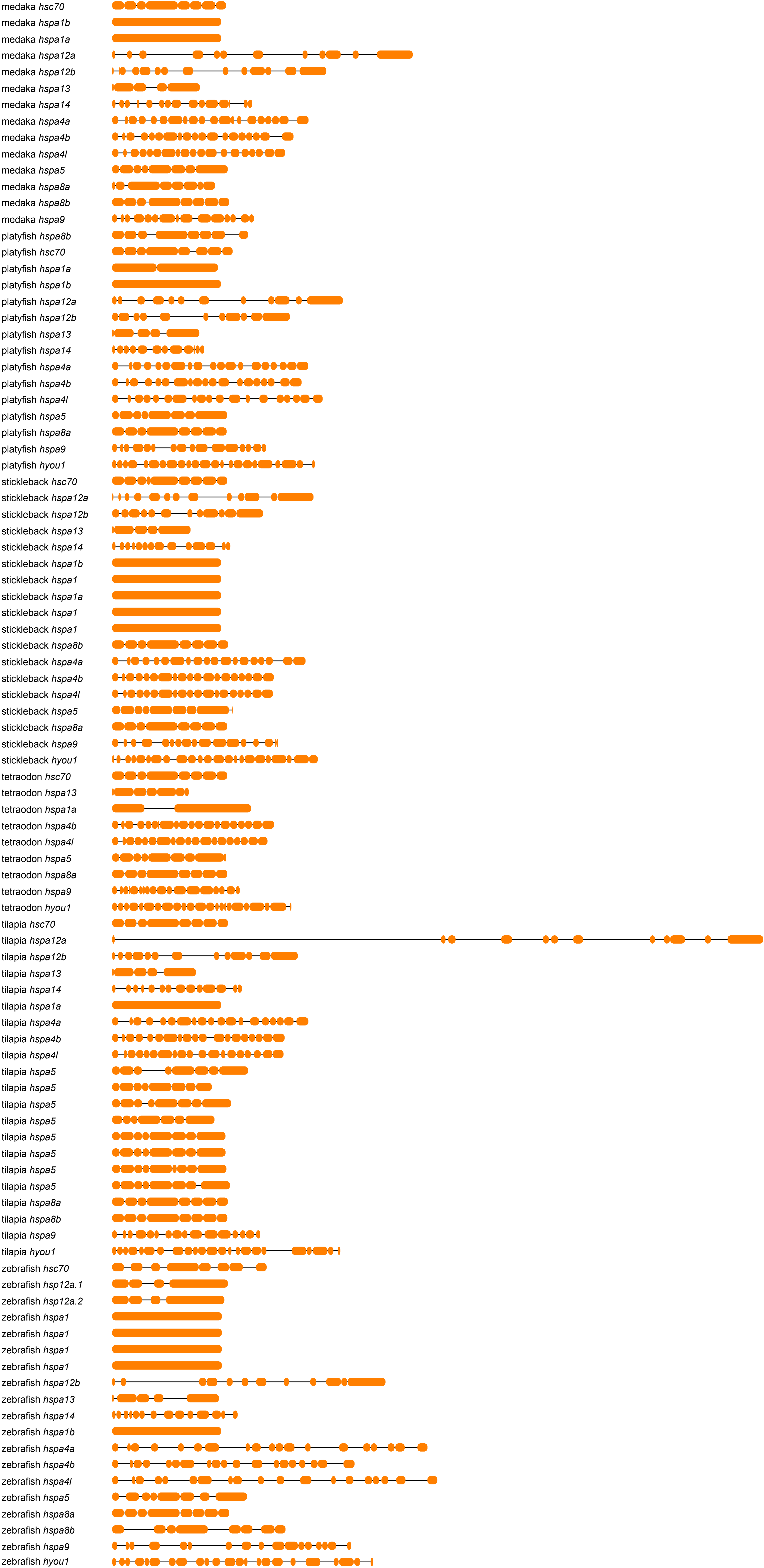

Legend: Exon Intron

Supplement: Figure S1 — Orange rectangles represent exons and black lines indicate introns, black polylines indicate introns, orange, and the black line indicates the scale. [file peerj-07-7781-s004.pdf]

# Motif 1

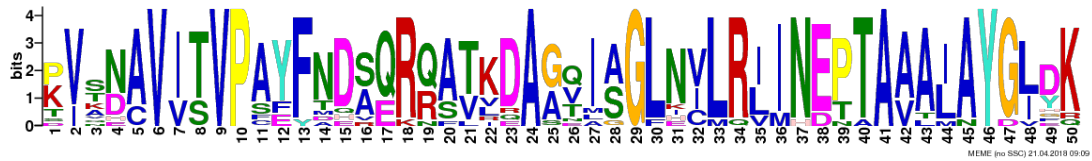

## Motif 2

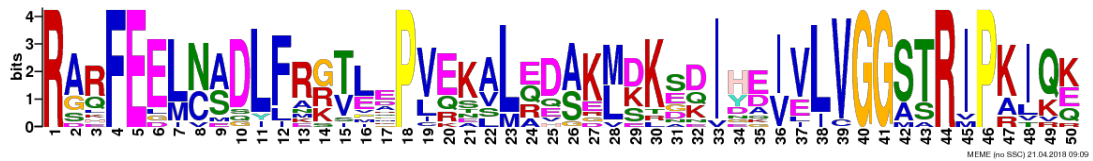

## Motif 3

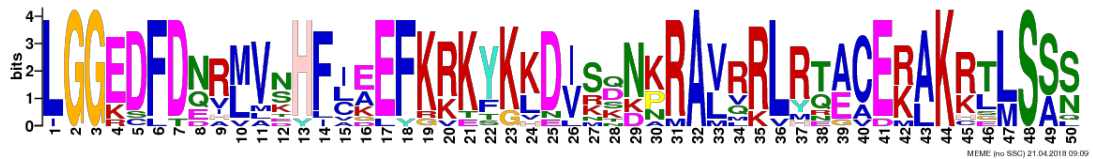

## Motif 4

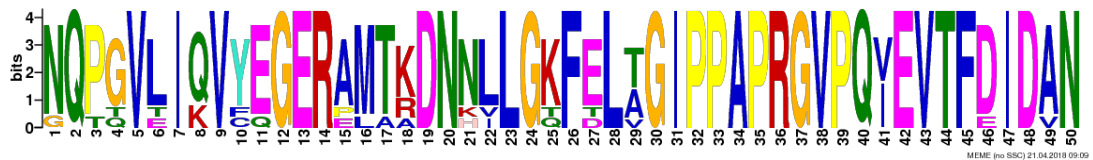

## Motif 5

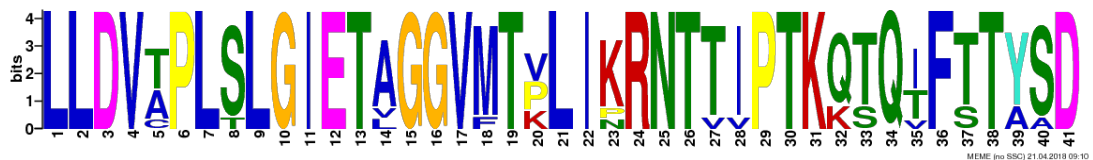

## Motif 6

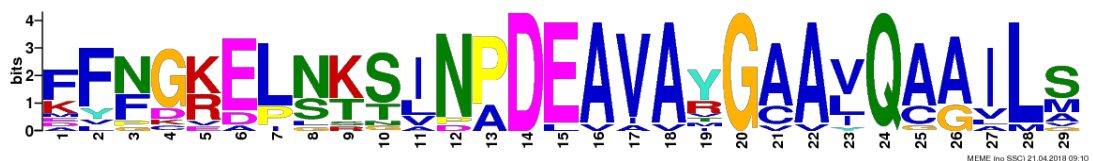

## Motif 7

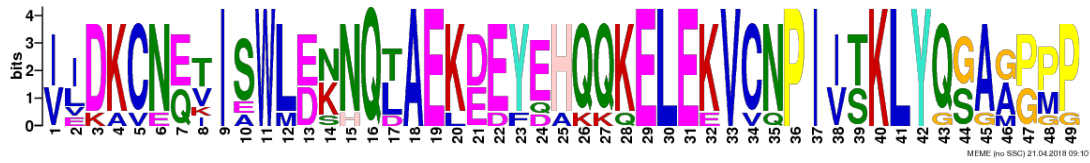

## Motif 8

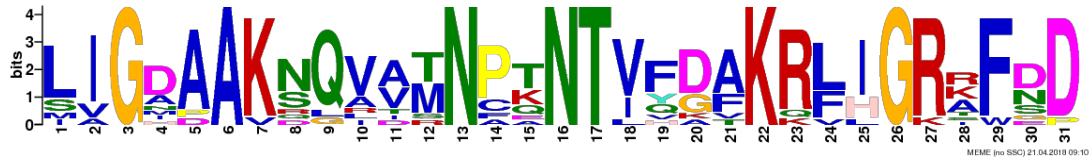

## Motif 9

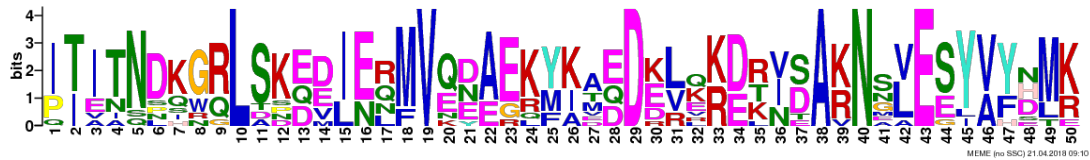

## Motif 10

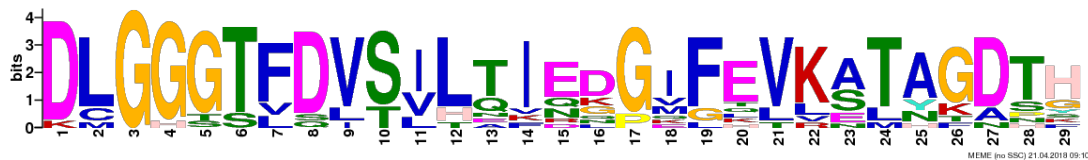

## Motif 11

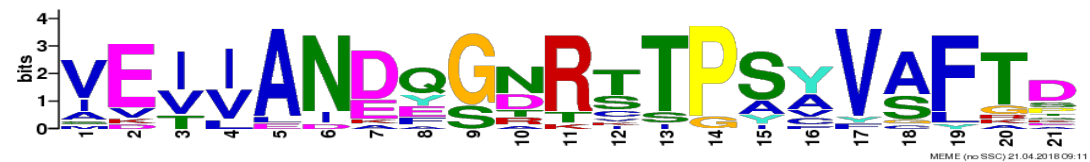

## Motif 12

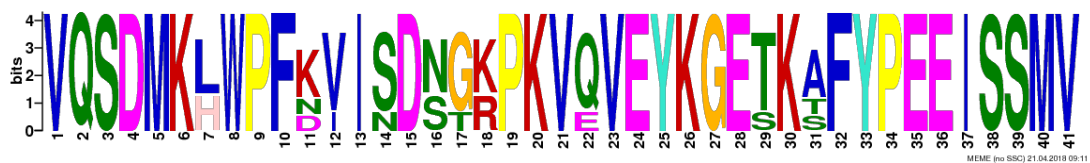

## Motif 13

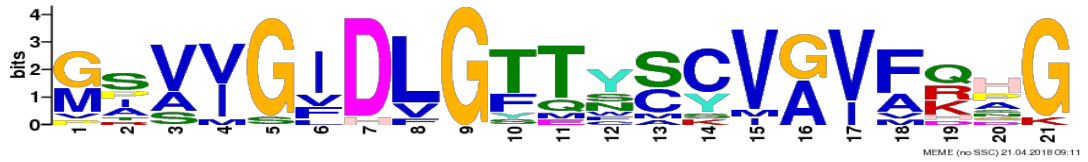

## Motif 14

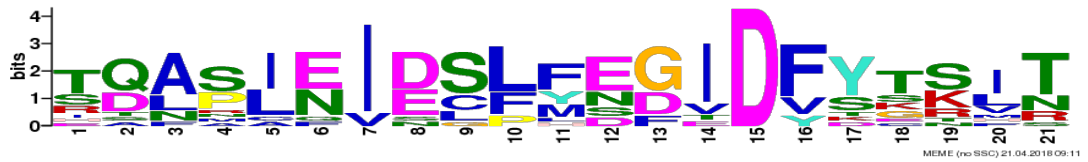

## Motif 15

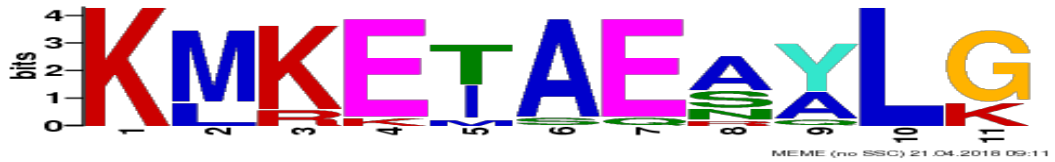

Supplement: Figure S2 — The motif corresponds to Fig. 3 of the body page. The same number inside the legend of Fig. 3 and this figure indicates the same motif. [file peerj-07-7781-s005.pdf]
